# TWIN SISTER OF FT (TSF) Interacts with FRUCTOKINASE6 and Inhibits Its Kinase Activity in Arabidopsis

**DOI:** 10.3389/fpls.2017.01807

**Published:** 2017-10-18

**Authors:** Suhyun Jin, Sun Young Kim, Ji Hoon Ahn

**Affiliations:** Department of Life Sciences, Korea University, Seoul, South Korea

**Keywords:** TSF, FT, FRK6, fructokinase, kinase activity, Arabidopsis

## Abstract

In flowering plants, the developmental switch to the reproductive phase is tightly regulated and involves the integration of internal and external signals. FLOWERING LOCUS T (FT) and TWIN SISTER OF FT (TSF) integrate signals from multiple pathways. FT and TSF function as florigenic substances, and share high sequence similarity with mammalian Raf kinase inhibitor protein (RKIP). Despite their strong similarity to RKIP, the kinase inhibitory activity of FT and TSF remains to be investigated. We performed a yeast two-hybrid screen and found that TSF interacted with FRUCTOKINASE6 (FRK6), which phosphorylates fructose for various metabolic pathways. Among the seven Arabidopsis FRKs, FRK6 and FRK7 have high sequence similarity; therefore, we investigated whether TSF interacts with FRK6 and FRK7. *In vitro* pull-down assays and bimolecular fluorescence complementation assays revealed that TSF interacts with FRK6 in the nucleus, but not with FRK7. Kinase activity assays suggested that TSF inhibits the kinase activity of FRK6, whereas FT does not. By contrast, neither TSF nor FT inhibits the kinase activity of FRK7. The *frk6* and *frk7* mutants show slightly delayed flowering, but only under short-day (SD) conditions. Plastochron length is also affected in both *frk6* and *frk7* mutants under SD conditions. *FT* expression levels decreased in *frk6* mutants, but not in *frk7* mutants. Taken together, our findings suggest that TSF physically interacts with FRK6 and affects its kinase activity, whereas FT does not, although these proteins share high sequence similarity.

## Introduction

Plants have evolved mechanisms that adjust their flowering time by integrating diverse internal or external signals (Srikanth and Schmid, 2011). Numerous genetic studies have revealed the interconnected pathways that control the floral transition in *Arabidopsis thaliana*, namely, the photoperiod, vernalization, gibberellic acid, autonomous, and ambient temperature pathways (Sung and Amasino, 2004; Corbesier et al., 2007; Lee et al., 2008; Wellmer and Riechmann, 2010). FLOWERING LOCUS T (FT), a well-known floral activator and a potential florigenic substance (Zeevaart, 2008; Putterill and Varkonyi-Gasic, 2016), acts as an integrator of the multiple signals that are transduced via various pathways and transmits the signals to trigger the onset of flowering.

Sucrose, a primary end product of photosynthesis, plays a pivotal role as the carbon source for most metabolic pathways (Rolland et al., 2002). Because sucrose is a disaccharide of glucose and fructose, it must be cleaved by invertase or sucrose synthase prior to its use as a substrate in metabolism (Sturm, 1999; Koch, 2004). The free hexoses generated by these sucrose-cleaving enzymes must be phosphorylated by specific kinases, such as fructokinase (FRK) and hexokinase (HXK), before entering the metabolic process (Smeekens, 2000). Hence, hexose-phosphorylating enzymes have essential functions for maintaining plant metabolism and development.

The hexose-phosphorylating enzyme FRK plays an important role in the production of functional metabolites. HXK also has fructose phosphorylating activity, but the affinity of HXK for fructose is much lower than that of FRK (Renz and Stitt, 1993). Among higher plants, the functions of FRKs are best characterized in tomato (*Solanum lycopersicum*). Tomato FRKs play a role in the development of vascular tissue and pollen (German et al., 2003). Furthermore, the suppression of *FRK1* via RNA interference caused delayed flowering in tomato (Odanaka et al., 2002). Consistent with the important roles of FRKs in plant development, plant genomes contain multiple *FRK* or *FRK*-like genes. In particular, the *A. thaliana* genome contains seven *FRK* genes. Arabidopsis *FRK6* and *FRK7* play a role in accumulation of seed storage proteins, and Arabidopsis *FRK1, FRK4, FRK6*, and *FRK7* are important for development of vascular tissue (Stein et al., 2017).

Arabidopsis FT/TSF family proteins are small globular proteins (approximately 175 amino acids) that play important regulatory roles in flowering. The *FT/TSF* genes include *FT, TWIN SISTER OF FT* (*TSF*), *TERMINAL FLOWER1, ARABIDOPSIS THALIANA CENTRORADIALIS HOMOLOG, MOTHER OF FT AND TFL1*, and *BROTHER OF FT AND TFL1* (Kardailsky et al., 1999; Kobayashi et al., 1999; Yoo et al., 2004; Yamaguchi et al., 2005; Huang et al., 2012). *TSF* has high sequence similarity to *FT*; their amino acid sequences are 82% identical, and TSF shows functional redundancy with FT. Overexpression of *TSF* or *FT* leads to extremely early flowering (Yamaguchi et al., 2005). Interestingly, the *tsf* mutants show strongly delayed flowering under short-day (SD) conditions, but the effect of the *tsf* mutation is very limited under long-day (LD) conditions (Yamaguchi et al., 2005). TSF plays a role in the promotion of flowering by cytokinin under non-inductive conditions (D’Aloia et al., 2011). These findings suggest that *TSF* plays an important role in the regulation of flowering time under SD conditions.

The FT/TSF family members were originally classified as phosphatidylethanolamine binding proteins. These proteins share strong amino acid sequence similarity with mammalian Raf kinase inhibitor protein (RKIP) (Schoentgen et al., 1987; Grandy et al., 1990; Bradley et al., 1996). In mammals, RKIP functions as a negative factor in Raf/MEK/ERK signaling, which helps ensure cell differentiation, growth, and survival in response to extracellular signals (Yeung et al., 1999, 2000). In the unstimulated state, RKIP associates with Raf and interferes with the phosphorylation activity of Raf for MEK/ERK (Corbit et al., 2003). Extracellular stimulus-induced phosphorylation of RKIP causes the release of Raf from RKIP, subsequently activating the MEK/ERK cascade (Corbit et al., 2003). Thus, it appears that RKIP is strongly linked to various physiological processes in higher organisms, from plants to mammals. As FT/TSF family proteins contain an evolutionarily conserved ligand-binding domain that is present in RKIP (Kardailsky et al., 1999), circumstantial evidence suggests that FT and TSF also function as kinase inhibitors in Arabidopsis. However, this potential function of these proteins has not been investigated.

In this study, we show that TSF, but not FT, interacts with FRK6 and inhibits its kinase activity. The *frk6* mutants showed slightly delayed flowering under SD conditions, which was attributed to reduction in *FT* expression. Our findings therefore suggest that TSF functions as a FRK inhibitor in Arabidopsis.

## Materials and Methods

### Plant Materials and Flowering Time Measurements

The *frk6-1* (SALK_143725), *frk6-2* (SALK_044085), and *frk7-2* (SALK_203384) mutants were obtained from the ABRC^1^ and were grown at 23°C. The T-DNA insertions in these mutants were confirmed via PCR genotyping using primers flanking the T-DNA (p1 and p2 for *frk6-1*, p3 and p4 for *frk6-2*, and p5 and p6 for *frk7-2*, Supplementary Table S1). Total leaf number and plastochron length were measured under both LD and SD conditions. Total leaf number was counted when the size of the primary inflorescence reached approximately 5 cm. Box plots were constructed to represent flowering time distribution (Williamson et al., 1989; Spitzer et al., 2014).

### Yeast Two-Hybrid Screening

The full-length *TSF* gene was cloned in the *Sma*I/*Sal*I sites of the pB2TK vector, which contains the DNA binding domain of GAL4. The junction of the GAL4 DNA binding domain and *TSF* was confirmed by sequencing. Screening was performed on 4.0 × 10^6^ colonies from an Arabidopsis whole seedling cDNA library. The yeast PBN204 strain containing three reporter genes (*URA3, lacZ*, and *ADE2*) under the control of different GAL promoters was used. Yeast cells transformed with the *TSF* bait vector and an Arabidopsis cDNA AD library were spread onto selection medium (SD-leucine, tryptophan, uracil [SD-LWU]), which supports the growth of yeast harboring bait and prey plasmids, yielding proteins that interact with each other. To confirm the interaction, the portions of prey DNA from URA3^+^, ADE2^+^, and lacZ^+^ candidates were amplified by PCR, and the resulting amplified prey sequences were re-introduced into yeast with the *TSF* bait plasmid. Yeast two-hybrid screening was conducted by PanBionet Corp. (Pohang, South Korea).

### Phylogenetic Analysis

Amino acid sequence alignment was performed using MUSCLE (Edgar, 2004). A phylogenetic tree was constructed using the maximum likelihood method implemented in the PhyML program of the software phylogeny.fr^2^ with default parameters (Guindon and Gascuel, 2003; Dereeper et al., 2008). The tree was visualized by using TreeDyn (Chevenet et al., 2006) with mid-point rooting.

### mRNA Expression Analyses

*FRK6* and *FRK7* mRNA levels were analyzed by semi-quantitative RT-PCR. *FT* expression was analyzed via qPCR. Total RNA was extracted from 5-day-old Arabidopsis seedlings sampled at ZT14 (unless otherwise indicated) using Plant RNA purification reagent (Invitrogen). The RNA (1 μg) was reverse transcribed into cDNA using a Transcriptor First Strand cDNA Synthesis kit (Roche). For qPCR, expression analysis was performed using SYBR Green I Master mix (Roche) in a LightCycler 480 (Roche). The data were normalized against two stable reference genes, *PP2AA3* (*AT1G13320*) and a *SAND* family gene (*AT2G28390*) (Hong et al., 2010). All qPCR data are presented as the mean of two biological replicates with three technical replicates each, and the error bars indicate the standard deviation. Statistical significance of differences in gene expression levels between the samples was assessed using Student’s *t*-test; differences at *P* < 0.05 were considered significant. Information about the primers used in this study is presented in Supplementary Table S1.

### Recombinant Protein Expression and Purification

To prepare His-tagged FRKs, the full-length *FRK6* (*At1g66430*) coding sequence (CDS) including a predicted chloroplast transit peptide (cTP) and the full-length *FRK7* (*At5g51830*) CDS were PCR-amplified and the products were cloned into the pET21a vector (EMD Biosciences). The recombinant constructs were introduced into *Escherichia coli* BL21 cells. After overnight culture at 28°C with 0.2 mM IPTG, the transformed cells were harvested and resuspended in lysis buffer (50 mM Tris–HCl pH 8.0, 300 mM NaCl, 20 mM imidazole, 2% N-lauroylsarcosine sodium salt). The lysates were collected after sonication and centrifugation and loaded onto a His Trap column (GE Healthcare). Further purification was performed according to the manufacturer’s instructions.

To prepare His-tagged FT and His-tagged TSF, the CDSs of *FT* (*At1g65480*) and *TSF* (*At4g20370*) were cloned into the pET28a vector after restriction enzyme digestion. *E. coli* strain BL21 cells transformed with each recombinant plasmid were grown at 28°C with 0.1 mM IPTG for induction. Protein purification was conducted using the lysis buffer and procedure described above.

### *In Vitro* GST Pull-down Assays

To prepare glutathione S-transferase (GST)-tagged TSF for the *in vitro* pull-down assays, the full-length CDS of *TSF* was cloned into the pGEX-5X-1 vector and introduced into *E. coli* BL21 cells. GST only or GST-tagged TSF was expressed in *E. coli* BL21 cells at 28°C with 0.15 mM IPTG. After the protein extracts were sonicated in GST lysis buffer (50 mM Tris–HCl pH 7.5, 0.1 M NaCl, 0.05% Tween-20, 1 mM EDTA pH 8.0, 1 mM PMSF, and protease inhibitor cocktail), the cell lysates were incubated in a glutathione-Sepharose 4B (GE healthcare) slurry for 1 h at 4°C and washed three times with the same lysis buffer.

For the *in vitro* pull-down experiment, purified His-tagged FRK6 and His-tagged FRK7 were incubated with equal amounts of GST only or GST-fused TSF immobilized on glutathione-Sepharose 4B beads for 1 h at 4°C. After the binding reaction, the beads were washed four times with GST lysis buffer. Proteins bound to beads were dissociated by adding SDS–PAGE sample buffer and loaded onto a 15% SDS–PAGE gel. Immunoblotting was performed using anti-His (Santa Cruz) or anti-GST (Santa Cruz) primary antibodies and goat anti-rabbit IgG secondary antibodies. The bands were visualized by applying Enhanced Chemiluminescence solution (AbClon).

### Bimolecular Fluorescence Complementation (BiFC) Assays

To generate the constructs used for the BiFC experiments, full-length *TSF, FRK6* (including a predicted cTP), and *FRK7* CDSs were PCR-amplified from cDNA prepared from wild-type plants. The PCR products were cloned in the *Bam*HI/*Xho*I sites of the pUC-SPYNE and pUC-SPYCE vectors, respectively. Protoplasts were isolated from 4-week-old Arabidopsis leaves as described previously (Yoo et al., 2007). Recombinant plasmids for BiFC containing N- and C-terminal YFP fragments were co-transfected into the protoplasts using the polyethylene glycol transformation method (Yoo et al., 2007). The transformed protoplasts were incubated for 12 h, and YFP signals were detected by confocal microscopy (Zeiss LSM700). bZIP63 (*At5g28770*) was used as a positive control for the BiFC experiments. bZIP63 was fused with N- and C-terminal YFP fragments; thus, YFP fluorescence was detected in the nucleus only if bZIP63 formed a homodimer. YFP and autofluorescence were excited at 513 nm and visualized at 530–590 nm and 650–710 nm, respectively.

### Fructokinase Enzyme Activity Staining Assays

The effect of TSF and FT on FRK enzyme activity was investigated using a previously described staining method (Harris and Hopkinson, 1976; Gonzali et al., 2001). Electrophoresis of 1 μg FRK6-His (or FRK7-His) or a mixture of 1 μg FRK6-His (or FRK7-His) and 1 μg His-TSF (or His-FT) was performed in a native PAGE gel. 2 nmol Raf1 kinase inhibitor I (Millipore 553003) and 1 μg of purified recombinant His-COP9 Signalosome 5A (His-CSN5a) were also used for the enzyme activity staining assay. A staining mixture in 1% agarose solution at concentrations suggested by Gonzali et al. (2001) was poured on top of the native gel. After the overlaying agarose gels solidified, the enzymatic reaction was conducted in the dark at room temperature for 1 h, followed by the addition of 1% acetic acid solution to stop the reaction. The intensity of formazan, the end product of the FRK reaction, was analyzed using ImageJ (Schneider et al., 2012).

## Results

### Yeast Two-Hybrid Screening Identifies FRK6 as an Interactor of TSF

**FIGURE 1:**
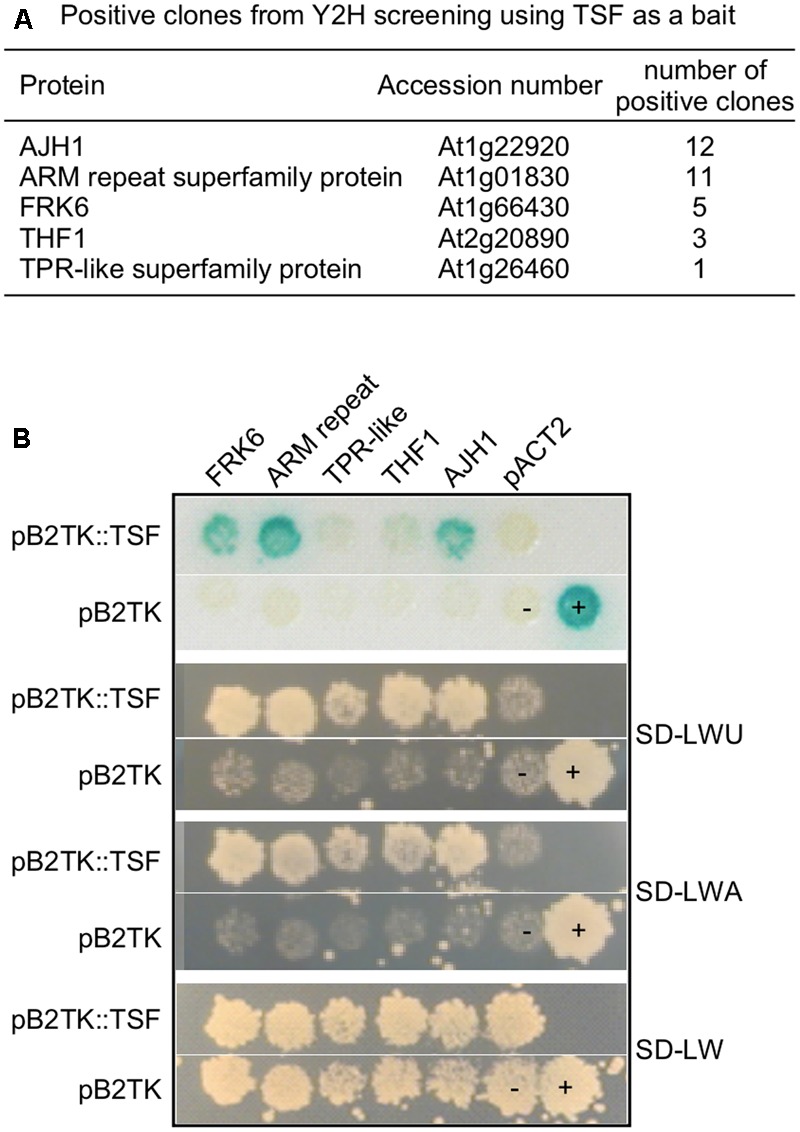
Yeast two-hybrid screening using TSF as a bait. **(A)** List of positive clones from yeast two-hybrid screening using TSF as a bait. **(B)** Interaction test of TSF interactors in yeast strain PBN204. SD-LWU is a selection medium lacking leucine (L), tryptophan (W), and uracil (U).

To identify interactors of TSF, we performed yeast two-hybrid screening using TSF as bait. Full-length TSF cloned in pGBKT shows self-transcriptional activity (data not shown); therefore, we cloned *TSF* in the pB2TK vector, which allows the bait protein to be expressed at lower levels. Among the 231 URA3^+^ colonies, 198 lacZ^+^ colonies, and 155 ADE2^+^ colonies obtained, we identified 60 colonies that were URA3^+^, ADE2^+^, and lacZ^+^. After confirming the interaction by reintroducing the amplified prey portion of DNA from the 60 URA3^+^, ADE2^+^, and lacZ^+^ candidates, we identified 32 positive clones (**Figures 1A,B**), including: JAB1 HOMOLOG 1 (AJH1; At1g22920), an armadillo/beta-catenin-like repeat-containing protein (ARM repeat superfamily protein; At1g01830), FRK6 (FRK6; At1g66430), THYLAKOID FORMATION 1 (THF1; At2g20890), and a tetratricopeptide repeat (TPR)-like superfamily protein (TPR-like superfamily protein; At1g26460). In the case of FRK6, the activation domain was fused to the 5′ UTR of *FRK6* and N-terminal 14 amino acids (from M1 to G14) were found to interact with TSF. Among these, we decided to investigate FRK6, because FT, the closest homolog of TSF, has sequence similarity to mammalian RKIP (Kardailsky et al., 1999). Therefore, we reasoned that analyzing the interaction between TSF and FRK might reveal a role in inhibition of kinase function.

### Phylogenetic Analysis of Arabidopsis FRKs

**FIGURE 2:**
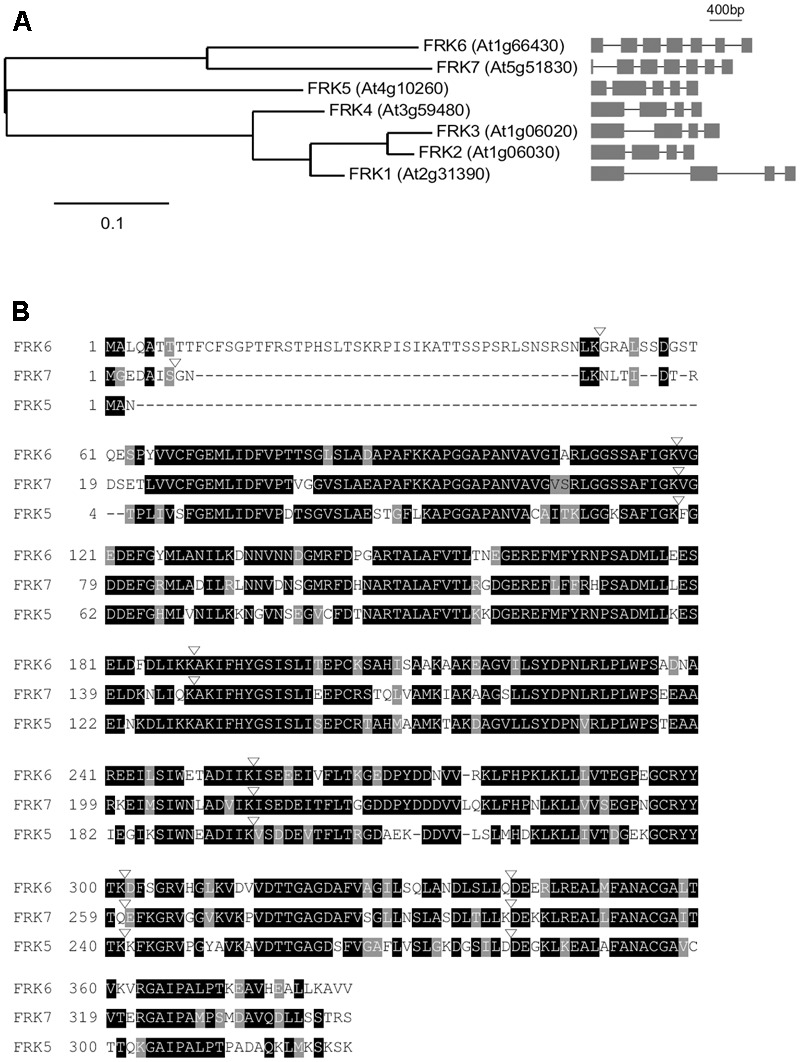
Sequence alignment of Arabidopsis FRKs. **(A)** Phylogenetic tree of Arabidopsis *FRK* genes. The phylogenetic tree was constructed using the maximum likelihood method with mid-point rooting (Guindon and Gascuel, 2003; Dereeper et al., 2008). Scale bar: the number of amino acid changes per site. The exon/intron structure of each fructokinase gene is shown on the right. **(B)** Alignment of FRK6 and FRK7 amino acid sequences using the T-Coffee Multiple Sequence Alignment tool. FRK5, which is the next related FRK, is included in this alignment to show that FRK6 and FRK7 are closely related. Black and gray shading indicate identical and conserved residues, respectively. Inverted triangles denote exon/intron boundaries. Dashes were introduced to maximize amino acid alignment.

Before analyzing the relationship between FRK6 and TSF, we analyzed the sequence similarity of the FRKs to identify any close homologs of *FRK6* in the Arabidopsis genome. The Arabidopsis genome contains seven *FRK* genes encoding proteins with fructose phosphorylating activity: *FRK1* (*At2g31390*), *FRK2* (*At1g06030*), *FRK3* (*At1g06020*), *FRK4* (*At3g59480*), *FRK5* (*At4g10260*), *FRK6* (*At1g66430*), and *FRK7* (*At5g51830*) (Stein et al., 2017). FRK1–7 contain 325, 329, 345, 326, 324, 384, and 343 amino acids. Notably, FRK6 contains additional 46 amino acids that were predicted as a cTP at its N-terminus. We classified the seven *FRK* genes according to evolutionary distances (**Figure 2A**). As shown in the phylogram, *FRK7* is more closely aligned with *FRK6* than with the five other *FRKs*, suggesting that *FRK6* and *FRK7* are homologs. Consistent with this notion, among the Arabidopsis FRK genes, only *FRK6* and *FRK7* have seven exons, whereas *FRK1–FRK5* have four or five exons. The amino acid sequences of FRK6 and FRK7 share 75.1% sequence similarity and 63.1% sequence identity (**Figure 2B**). Furthermore, the exon/intron boundaries of FRK6 and FRK7 are also conserved. We reasoned that TSF might also interact with FRK7; thus, we used both FRK6 and FRK7 for further protein–protein interaction analyses.

### Protein–Protein Interactions between TSF and FRK6

**FIGURE 3:**
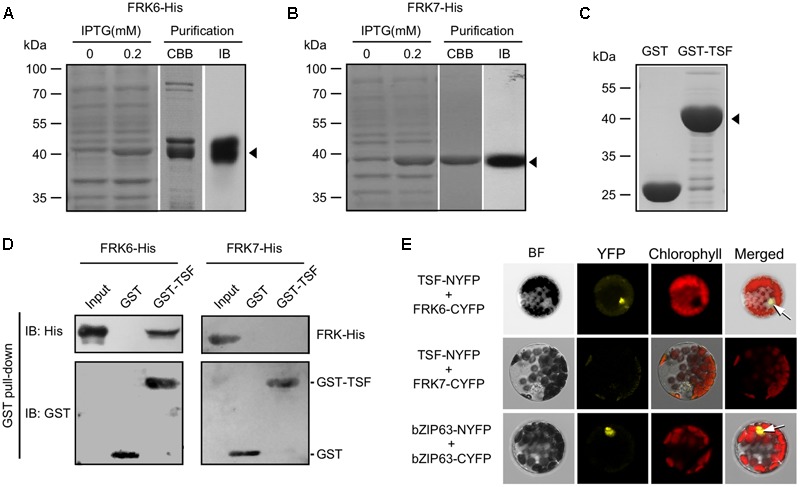
Interaction between TSF and FRK6. **(A,B)** Purification of FRK6-His **(A)** and FRK7-His **(B)** expressed in *E. coli* using a His column. Immunoblotting was performed using anti-His antibodies to confirm the purity of the FRK-His proteins (arrowhead). CBB: Coomassie brilliant blue staining, IB: immunoblot **(C)** Immobilized GST only or GST-TSF on glutathione-Sepharose 4B beads (arrowhead). **(D)** *In vitro* pull-down assays using GST-TSF and FRK6-His/FRK7-His. Note that GST-TSF co-precipitated with FRK6-His, but not with FRK7-His. **(E)** BiFC assays showing that TSF interacts with FRK6 and that this complex localizes to the nucleus (upper arrow). bZIP63 was used as a positive control for protein–protein interaction in the nucleus (lower arrow). BF: bright field.

To test the interaction between TSF and FRK6/FRK7, we performed *in vitro* pull-down assays. We expressed FRK6 and FRK7 proteins with a 6X His tag in *E. coli* as a prey for the pull-down experiments, followed by purification through a His column (**Figures 3A,B**). Following gel electrophoresis of purified FRK6-His protein, Coomassie brilliant blue staining of the gel revealed additional minor bands near the putative FRK6-His protein. We therefore performed immunoblot analysis using anti-His antibody to confirm that the purified product contained FRK6-His protein. Anti-His antibody successfully detected FRK6-His protein at the expected size (∼42 kDa) after blotting (**Figure 3A**, right panel). We also induced the production of FRK7-His under the same conditions used for FRK6-His (**Figure 3B**, left panel). FRK7-His was highly enriched in the purification eluate, as shown by Coomassie brilliant blue staining and immunoblot analysis (**Figure 3B**, right panel). To prepare the bait protein for the pull-down assays, we expressed TSF with a GST tag in *E. coli* and immobilized the protein onto glutathione-Sepharose 4B beads (**Figure 3C**). The purified GST-TSF and FRK6-His/FRK7-His proteins were used for pull-down assays.

Our *in vitro* pull-down assays revealed that although GST and GST-TSF were present in almost equal amounts, GST-TSF bound to FRK6-His and was detected in the co-precipitated fraction via immunoblot analysis (**Figure 3D**). However, immunoblot analyses using anti-His antibody did not detect any co-precipitating FRK7-His. These results suggest that GST-TSF interacted with FRK6-His but not FRK7-His. Neither FRK6-His nor FRK7-His interacted with GST alone. These results suggest that TSF interacts with FRK6, but not with FRK7, *in vitro*.

To further validate the TSF–FRK6 protein interaction, we conducted BiFC assays. We co-transfected encoding TSF fused with the N-terminal fragment of YFP and FRKs fused with the C-terminal fragment of YFP into protoplasts; bZIP63 fused with N-terminal and C-terminal YFP fragments was included as a positive control for protein–protein interactions. YFP signal was only observed in the nucleus of protoplasts co-expressing TSF-NYFP and FRK6-CYFP (**Figure 3E**), suggesting that our *in vitro* GST pull-down results were reproduced in the BiFC assays. However, no fluorescent signal was detected in protoplasts co-transfected with TSF-NYFP and FRK7-CYFP, although we confirmed TSF-NYFP and FRK7-CYFP expression in the co-transfected protoplast via a western blot analysis (Supplementary Figure S1). It suggested that TSF does not interact with FRK7. Protoplasts co-expressing bZIP63-NYFP and bZFIP63-CYFP (positive control) showed fluorescent signals in the nucleus. Therefore, our GST pull-down and BiFC results suggest that TSF directly interacts with FRK6, but not with FRK7.

### TSF Inhibits the Phosphorylation of Fructose by FRK6

**FIGURE 4:**
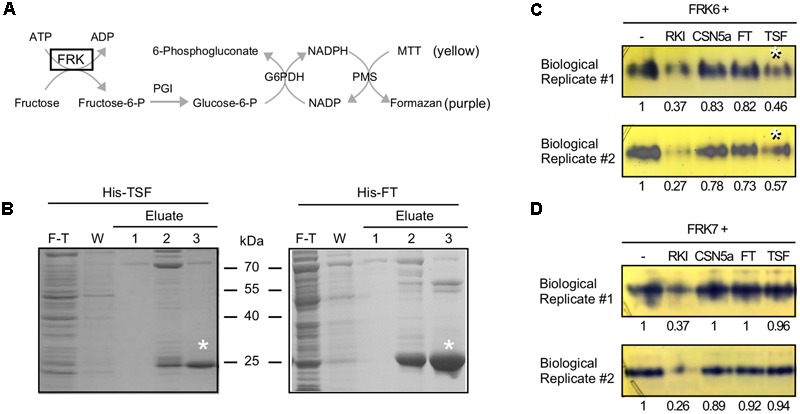
TSF inhibits FRK6 activity *in vitro.* **(A)** Schematic diagram of the enzyme assay used to measure FRK6 and FRK7 activity in this study. If active fructokinase is present in the reaction mixture, MTT (yellow) is converted into formazan (purple); however, if FRK activity is inhibited, the production of the purple compound is reduced. G6PDH: glucose-6-phosphate dehydrogenase; MTT: 3-(4,5-dimethylthiazol-2-yl)-2,5-diphenyltetrazolium bromide; PGI: phosphoglucoisomerase; PMS: phenazine methosulfate **(B)** Purification of His-TSF and His-FT proteins for enzyme activity assays. Asterisks indicate purified His-TSF and His-FT proteins. F-T: flow-through, W: wash **(C,D)** The effect of TSF on FRK6 **(C)** and FRK7 **(D)** activity. The numbers below each band indicate the fold-change relative to the formazan level under FRK6-His treatment or FRK7-His treatment only. Note that the production of formazan is reduced by His-TSF (**C**, asterisks), suggesting that TSF inhibits the activity of FRK6. By contrast, His-FT protein does not inhibit formazan production. Neither His-TSF nor His-FT inhibits FRK7 activity **(D)**. RKI: Raf1 kinase inhibitor I.

After confirming of the binding of TSF to FRK6, we performed an enzyme activity staining assay to investigate whether TSF inhibits the kinase activity of FRK6 (Harris and Hopkinson, 1976; Gonzali et al., 2001). The basic principle of this method is shown in **Figure 4A**. If active FRK is contained in the reaction mixture, it phosphorylates fructose to fructose-6-p, which phosphoglucose isomerase (PGI) converts into glucose-6-p, the primary substrate of the staining reaction. When the primary substrate is produced via FRK, the downstream reactions occur consecutively in the reaction mixture. Ultimately, the 3-(4,5-dimethylthiazol-2-yl)-2,5-diphenyltetrazolium bromide (MTT) in the mixture is reduced to the purple compound formazan; thus, FRK enzyme activity can be measured by analyzing the intensity of formazan staining in the native PAGE gel. We prepared His-TSF and His-FT proteins for this assay (**Figure 4B**), along with purified FRK6 and FRK7 fused with a 6X His tag.

To investigate whether TSF reduces FRK6 activity, we incubated FRK6-His with His-TSF; FRK6-His protein was also combined with Raf1 kinase inhibitor I and His-CSN5a proteins (Gusmaroli et al., 2004). Purple formazan staining was observed in the lane containing only FRK6-His (**Figure 4C**), indicating that the purified FRK6-His protein was functional. The intensity of formazan staining was reduced by the addition of Raf1 kinase inhibitor I, indicating that Raf1 kinase inhibitor I inhibits the activity of FRK6. However, the addition of His-CSN5a did not affect the activity of FRK6. As shown in **Figure 4C**, the formation of formazan was reduced approximately twofold by the addition of His-TSF in two biological replicates, whereas no reduction in formazan level was detected after the addition of His-FT. These results suggest that only His-TSF inhibits FRK6 enzymatic activity. By contrast, the addition of His-TSF to FRK7-His did not reduce the formation of formazan in both biological replicates (**Figure 4D**). Finally, the addition of His-FT to FRK7-His also failed to affect the formation of formazan. These results suggest that TSF inhibits the fructose phosphorylating activity of FRK6 via a direct physical interaction.

### *frk6* Mutants Show Late Flowering under SD Conditions

**FIGURE 5:**
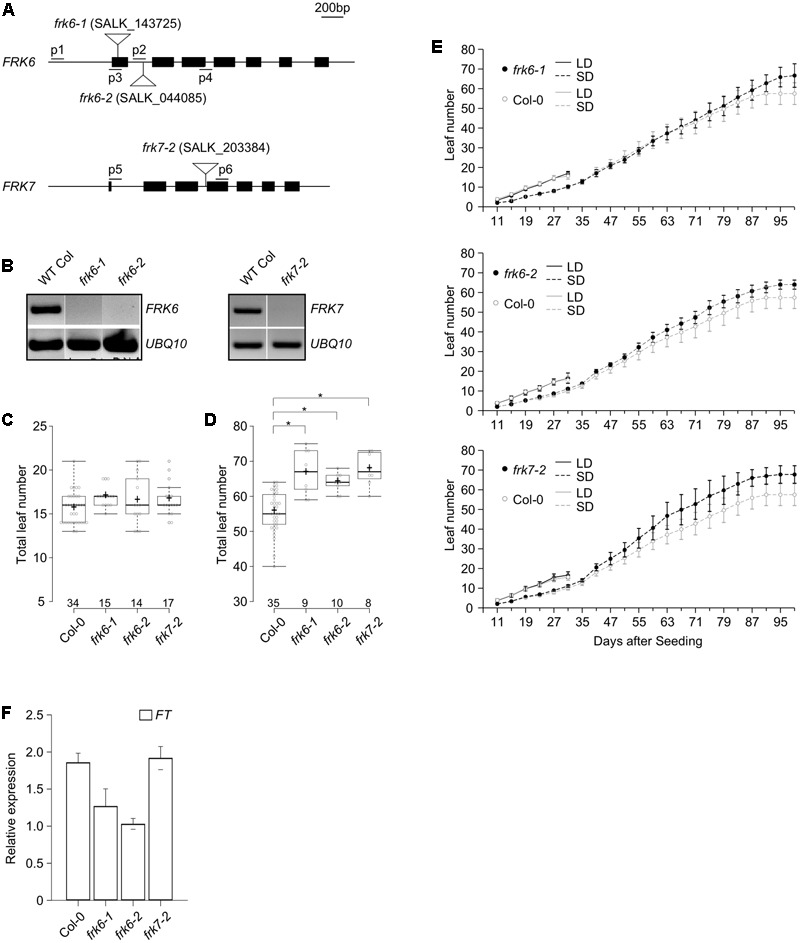
A late flowering phenotype is observed in the *frk6* and *frk7* mutants under SD conditions. **(A)** Map of the T-DNA insertion in the *frk6* and *frk7* mutants. Closed boxes indicate exons, and inverted triangles indicate the location of the T-DNA insertion site. The locations of genotyping primers (p1–p6) are shown. **(B)** *FRK6* or *FRK7* mRNA expression is absent in 7-day-old *frk6* and *frk7* plants grown under LD conditions. *UBIQUITIN10* (*UBQ10*) was used as an internal control. **(C,D)** Box plots showing total leaf number in *frk6* and *frk7* plants grown under LD **(C)** and SD conditions **(D)** at 23°C. Individual data points are displayed as circles in the box plot. The center lines show the medians, and plus signs (+) show the mean value; box limits indicate the 25th and 75th percentiles, as determined with R software; whiskers extend to 1.5-times the interquartile range (IQR) from the 25th and 75th percentiles, and outliers that exceed the 1.5X IQR are represented by ovals. The number of plants measured is shown above each genotype in the box plot. A *t*-test was used to assess the statistical significance of differences in flowering time observed in each mutant **(D)**. Asterisk: *p* < 0.001 **(E)** Plastochron length in *frk6* and *frk7* plants under LD and SD conditions at 23°C. Wild-type Columbia plants (control) are shown in both panels. **(F)** Expression levels of *FT* in *frk6* and *frk7* plants under SD conditions.

We next investigated whether the mutation of *FRKs* has a visible effect on the plant. To investigate the effect of *FRK6* and *FRK7* on flowering time, we obtained the *frk6* (SALK_143725 and SALK_044085) and *frk7* (SALK_203384) T-DNA mutants from the ABRC. SALK_143725 and SALK_044085 contain a T-DNA insertion in the first exon and first intron of *FRK6*, respectively (**Figure 5A**, top). SALK_203384 contains a T-DNA insertion at the end of the second intron of *FRK7* (**Figure 5A**, bottom). We confirmed the T-DNA insertions via PCR-genotyping using primers flanking both sides of the T-DNA (data not shown). *FRK6* and *FRK7* expression was severely affected by the T-DNA insertion in the mutants (**Figure 5B**), suggesting that these mutants are loss-of-function alleles of *FRK6* and *FRK7*. We therefore named SALK_143725, SALK_044085, and SALK_203384 as *frk6-1, frk6-2*, and *frk7-2*, respectively, and subjected these alleles to further analyses.

We measured flowering time and plastochron length in the *frk6* and *frk7* mutants under both LD and SD conditions at 23°C. None of the mutants showed visible differences in flowering compared to wild type under LD conditions (**Figure 5C**); *frk6-1, frk6-2*, and *frk7-2* mutants flowered when the plants had 17.0, 16.5, and 16.6 leaves, respectively, whereas wild-type plants flowered when they had 15.6 leaves under the same conditions. However, under SD conditions, both *frk6* and *frk7* mutants showed a slight but significant delay in flowering compared to wild type (**Figure 5D**). Under SD conditions, *frk6-1, frk6-2*, and *frk7-2* mutants flowered when they had 66.7, 64.0, and 67.8 leaves, whereas wild-type plants flowered when they had 55.7 leaves under the same conditions. Consistent with their altered flowering time, all mutants showed a slightly reduced plastochron length (increased leaf initiation rate) under SD conditions, which was more apparent in *frk7-2* mutants (**Figure 5E**). These observations suggest that the *FRK6* play a role in regulating flowering time under SD conditions.

Because the *frk6* and *frk7* mutants showed delayed flowering under SD conditions, we investigated the expression levels of flowering time genes in these plants via qPCR. Under SD conditions, *FT* mRNA levels were reduced only in *frk6-1* and *frk6-2* mutants (**Figure 5F**), whereas *FT* mRNA levels were not altered in *frk7-2* mutants. These results suggest that the late flowering phenotype of *frk6-1* and *frk6-2* mutants could be attributed to reduced *FT* expression levels under SD conditions.

## Discussion

In this study, we investigated whether TSF functions as a kinase inhibitor. We detected protein–protein interactions between TSF and FRK6 via *in vitro* pull-down and BiFC assays. TSF likely inhibits the fructose-phosphorylating activity of FRK6 via physical interaction. We also found that the *frk6* mutation affects the expression of *FT*, which appears to cause delayed flowering under SD conditions.

Although structural similarities suggest that FT and TSF, as well as their homologs play similar roles to that of mammalian RKIP (Kardailsky et al., 1999; Yeung et al., 1999), their potential roles as kinase inhibitors had not been investigated in plants. In this study, we showed that TSF binds to FRK6 (**Figures 1, 3**) and inhibits its activity (via an enzyme activity staining assay) (**Figure 4**). Arabidopsis FRK6 has high sequence similarity to FRK7 (**Figure 2**); however, TSF inhibits the activity of FRK6, but not FRK7, suggesting that the interaction between TSF and FRK6 is specific. Another interesting observation is that although TSF is homologous to FT, FT does not inhibit the activity of FRK6 or FRK7. All Arabidopsis FRKs except FRK1 exhibit substrate inhibition (Riggs et al., 2017), and FRKs are thought to play a role in regulating starch synthesis via sucrose synthase in the sink tissue of plants (Odanaka et al., 2002). Therefore, it would be interesting to further investigate a possible role for TSF in sink tissue.

According to our BiFC assay results, TSF likely interacts with FRK6 in the nucleus (**Figure 3E**), which is inconsistent with the results of a previous report (Stein et al., 2017). The majority of FRKs in tomato and Arabidopsis localize to the cytosol, except for tomato FRK3 (LeFRK3) and Arabidopsis FRK6, which localize to the plastid (Damari-Weissler et al., 2006; Riggs et al., 2017). Perhaps FRK6 interacts with TSF only in the nucleus, although FRK6 may localize to both the cytosol and nucleus. Indeed, our confocal microscopy analyses showed that FRK6-GFP signal was seen in the nucleus as well as chloroplasts, whereas GFP-TSF signal was observed in the nucleus (Supplementary Figure S2). However, unlike FRK6 and TSF, GFP-FRK7 signal was found in the cytosol. Thus, although FRK6 mainly localizes to the cytosol, a small fraction of FRK6 may be present in the nucleus, where it might interact with TSF. Consistent with this notion, Arabidopsis HXK1, another hexose-phosphorylating enzyme, localizes to the nucleus, where it forms a distinct protein complex with its interactors (Cho et al., 2006). Thus, it is tempting to speculate that nucleus-localized FRK6 directly interacts with TSF, which may be required for its novel nuclear-specific function.

Although a previous report suggested that *frk6* single mutants have no apparent mutant phenotype (Stein et al., 2017), we observed a slight delay in flowering time in the *frk6* mutants under SD conditions (**Figure 5**). This late flowering is likely due, at least in part, to the reduced levels of *FT* mRNA in these mutants. Mutants with impaired functioning in both FRK6 and FRK7 exhibit an altered seed phenotype (Stein et al., 2017), suggesting that they act redundantly in seed development. However, we found that both the *frk6* and *frk7* mutants showed a visible flowering time phenotype under SD conditions. This raised the possibility that the TSF–FRK6 module might play a role in modulating the juvenile-to-adult phase transition, as was observed for *TFL1* (Matsoukas et al., 2013).

The observations that TSF inhibits FRK6 activity (**Figure 4C**) and that impaired *FRK6* function caused late flowering under SD conditions (**Figure 5D**) appear to be inconsistent with the known role of TSF as a floral activator (Yamaguchi et al., 2005). A possible scenario to explain this discrepancy is as follows: Although TSF inhibits FRK6, which subsequently delays flowering, the inductive effect on flowering caused by the translocation of TSF to the shoot apical meristem is much stronger and overrides the effect of the *frk6* mutation on flowering. Indeed, like FT, TSF likely moves toward the shoot apical meristem to trigger flowering under non-inductive conditions (Corbesier et al., 2007; Jin et al., 2015). Thus, the promotive effect of long-distance movement of TSF likely overrides the effect of *frk* mutation under SD conditions. Another possible scenario is that the TSF-FRK6 module acts in tissues that are not involved in flowering, for instance, in sink tissue such as seeds (Stein et al., 2017). Further investigation is required to clarify the molecular mechanism underlying the activity of the TSF–FRK6 module in plants.

An important question is how potential alteration of carbon assimilation caused by *frk6* mutation is connected to the changes in flowering time. A possible scenario is that the changes in carbon assimilates partitioning by the *frk6* mutation does not play a main role in the regulation of flowering time, unlike FRK7; rather, FRK6 plays a role in the transcriptional regulation of downstream flowering time genes. Consistent with this notion, HXK, a hexose-phosphorylating enzyme, regulates the developmental transition via miR156. The level of miR156, which plays a pivotal role in the transition from the juvenile to the adult phase, is affected by *HXK1* in response to sugar (Yang et al., 2013; Yu et al., 2013). Nuclear-localized HXK1 directly or indirectly regulates miR156 expression via association with nuclear factors, for instance, VHA-B1 and RPT5B (Cho et al., 2006). The effect of HXK1 on the miR156 changed the levels of *SQUAMOSA-promoter binding protein-like* (*SPL*) genes (Wang et al., 2008), thereby affecting *FT* transcription (Kim et al., 2012). Notably, we found that FRK6 locates not only to the chloroplast but also to the nucleus, as seen in HXK1; furthermore, the *frk6* mutation caused reduction of *FT*. It is tempting to speculate that FRK6 may regulate the expression of the miR156-SPL module to eventually regulate *FT* transcript levels to control flowering time.

In summary, we identified a possible biochemical role of TSF as an inhibitor of FRK6 in plants. FT/TSF family members participate in various fundamental developmental processes in plants; however, no molecular evidence for their role as kinase inhibitors had previously been obtained, despite their homology to an animal kinase inhibitor protein. Our results open new avenues for investigating the biochemical functions of FT/TSF family proteins.

## Author Contributions

SJ and SYK performed the experiments. JHA designed and supervised the study. SJ and JHA wrote the manuscript.

## Conflict of Interest Statement

The authors declare that the research was conducted in the absence of any commercial or financial relationships that could be construed as a potential conflict of interest.

## Footnotes

1 http://abrc.osu.edu

2 http://www.phylogeny.fr/alacarte.cgi
