# Supplementary material for: TWIN SISTER OF FT (TSF) Interacts with FRUCTOKINASE6 and Inhibits Its Kinase Activity in Arabidopsis
Source: Front Plant Sci. 2017 Oct 18;8:1807. doi: 10.3389/fpls.2017.01807 (PMC5651264; doi:10.3389/fpls.2017.01807)
Supplement: Supplementary file 1 [file Table_1.DOCX]

Supplementary Material

**TWIN SISTER OF FT (TSF) Interacts with FRUCTOKINASE6 and Inhibits its Kinase Activity in Arabidopsis**

**Suhyun Jin^1^, Sun Young Kim^1^, and Ji Hoon Ahn^1*^**

*** Correspondence:**Ji Hoon Ahn, Ph.D.

Professor

Department of Life Sciences,

Korea University

Anamro 145, Seongbuk-Gu,

Seoul 02841

South Korea

Phone: 82-2-3290-3451

Fax: 82-2-927-9028

Email: jahn@korea.ac.kr

# Supplementary Table

- **Supplementary Table 1.** Oligonucleotide sequences used in this study

| Primer | Gene | | Oligonucleotide sequence (5′-> 3′) | Purpose |
| --- | --- | --- | --- | --- |
| JH9217 | *FRK6* | CGGACTTGTGCTCTTCACCT | | Genotyping (p1) |
| JH9218 | *FRK6* | GATCGTGAATTGGAGAGTCTTGA | | Genotyping (p2) |
| JH9215 | *FRK6* | CCAACTTTCCGATCAACTCCTC | | Genotyping (p3) |
| JH9216 | *FRK6* | TCAGCACTCGGGTTTCGG | | Genotyping (p4) |
| JH9914 | *FRK7* | TGGGTTTTTCGTTTTCATGG | | Genotyping (p5) |
| JH9915 | *FRK7* | AGACTCCCTGCAGCCTTAGC | | Genotyping (p6) |
| JH9520 | *FRK6* | ATGGCTCTCCAAGCCACTAC | | RT-PCR |
| JH9521 | *FRK6* | AACGACGGCTTTGAGTAGAG | | RT-PCR |
| JH9522 | *FRK7* | ATGGGTGAGGATGCAATCTC | | RT-PCR |
| JH9523 | *FRK7* | GGAGCGAGTAGAAGAAAGAA | | RT-PCR |
| JH6505 | *PP2A* | GCGGTTGTGGAGAACATGATACG | | qPCR |
| JH6506 | *PP2A* | GAACCAAACACAATTCGTTGCTG | | qPCR |
| JH7588 | *SAND* | TTGATCCACTTGCAGACAAGGC | | qPCR |
| JH7589 | *SAND* | TACCCTTTGGCACACCTGATTG | | qPCR |
| JH6350 | *FT* | CTGGAACAACCTTTGGCAAT | | qPCR |
| JH6351 | *FT* | AGCCACTCTCCCTCTGACAA | | qPCR |

**Supplementary Methods**

**Immunoblotting**

TSF-NYFP and FRK7-CYFP co-transfected cells were prepared as described in main text. After incubation for 12 hours, we harvested the protoplast cells via centrifugation at 200 x g for 2 min. Expressed proteins were extracted using the lysis buffer (25 mM Tris-HCl pH7.5, 150 mM NaCl, 0.5% Triton X-100, 1 mM EDTA, and protease inhibitor cocktail). TSF-NYFP and FRK7-CYFP were detected by using anti-c-myc (Santa Cruz) or anti-HA (Santa Cruz) antibody.

**Subcellular localization**

To generate GFP-tagged TSF, FRK6, and FRK7, full-length of *TSF* and *FRK7* were cloned into the 326-GFP vector via *Sma*I/*Xho*I restriction enzyme digestion. The CDS of *FRK6* including a predicted cTP was inserted into the 326-GFP vector after *Xba*I*/Bam*HI enzyme digestion. Each plasmid construct was transfected into the Col-0 protoplasts as described previously (Yoo et al., 2007). Fluorescent signal derived from NLS-RFP was used as the nuclear marker. Expressed fluorescence signals were observed under confocal microscopy (Zeiss LSM700). GFP, RFP, and chlorophyll were visualized at 580nm, 620nm, and 640nm, respectively, with excitation at 430nm.

**Reference**

Yoo, S.D., Cho, Y.H., and Sheen, J. (2007). Arabidopsis mesophyll protoplasts: a versatile cell system for transient gene expression analysis. *Nat Protoc* 2(7)**,** 1565-1572. doi: 10.1038/nprot.2007.199.

**FIGURE S1 | Confirmation of TSF-NYFP and FRK7-CYFP expression in co-transfected protoplasts**

**(A)** Schematic representation of BiFC construction of TSF-NYFP and FRK7-CYFP. **(B)** Western blot analysis using anti-HA or anti-c-myc antibody. TSF-NYFP and FRK7-CYFP were expressed in the co-transfected cells.

**FIGURE S2 | Subcellular localization of TSF, FRK6, and FRK7**

Subcellular localization of GFP-TSF, FRK6-GFP, and GFP-FRK7. RFP fused with nuclear localization signal (NLS) was co-transfected to show the location of the nucleus.
