# Supplementary figures and images for: TWIN SISTER OF FT (TSF) Interacts with FRUCTOKINASE6 and Inhibits Its Kinase Activity in Arabidopsis
Source: Front Plant Sci. 2017 Oct 18;8:1807. doi: 10.3389/fpls.2017.01807 (PMC5651264; doi:10.3389/fpls.2017.01807)

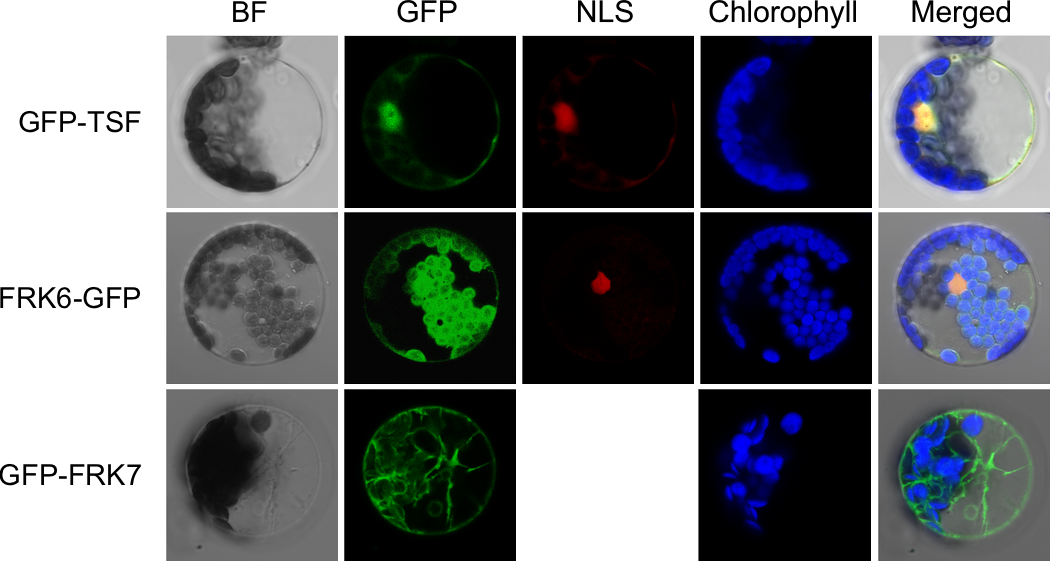

Supplement: Supplementary file 3 [file Image_2.TIF]
